# The Michigan Genetic Hereditary Testing (MiGHT) study’s innovative approaches to promote uptake of clinical genetic testing among cancer patients: a study protocol for a 3-arm randomized controlled trial

**DOI:** 10.1186/s13063-023-07125-2

**Published:** 2023-02-10

**Authors:** Lynette Hammond Gerido, Jennifer J. Griggs, Ken Resnicow, Kelley M. Kidwell, Emerson Delacroix, Sarah Austin, Erika N. Hanson, Elizabeth Bacon, Erika Koeppe, Stefanie Goodall, Matthew Demerath, Elizabeth A. Rizzo, Shayna Weiner, Sarah T. Hawley, Wendy R. Uhlmann, J. Scott Roberts, Elena M. Stoffel

**Affiliations:** 1 University of Michigan School of Public Health, Ann Arbor, USA; 2 University of Michigan Rogel Cancer Center, Ann Arbor, USA; 3 University of Michigan School of Medicine, Ann Arbor, USA; 4 Department of Internal Medicine, University of Michigan, Ann Arbor, USA

**Keywords:** RCT, Genetic testing, Genetic counseling, Cancer, Motivational interviewing, eHealth

## Abstract

**Background:**

Although most cancers are sporadic, germline genetic variants are implicated in 5–10% of cancer cases. Clinical genetic testing identifies pathogenic germline genetic variants for hereditary cancers. The Michigan Genetic Hereditary Testing (MiGHT) study is a three-arm randomized clinical trial that aims to test the efficacy of two patient-level behavioral interventions on uptake of cancer genetic testing.

**Methods:**

The two interventions being tested are (1) a virtual genetics navigator and (2) motivational interviewing by genetic health coaches. Eligible participants are adults with a diagnosis of breast, prostate, endometrial, ovarian, colorectal, or pancreatic cancer who meet the National Comprehensive Cancer Network (NCCN) criteria for genetic testing. Participants are recruited through community oncology practices affiliated with the Michigan Oncology Quality Consortium (MOQC) and have used the Family Health History Tool (FHHT) to determine testing eligibility. The recruitment goal is 759 participants, who will be randomized to usual care or to either the virtual genetics navigator or the motivational interviewing intervention arms. The primary outcome will be the proportion of individuals who complete germline genetic testing within 6 months.

**Discussion:**

This study addresses patient-level factors which are associated with the uptake of genetic testing. The study will test two different intervention approaches, both of which can help address the shortage of genetic counselors and improve access to care.

**Trial registration:**

This study has been approved by the Institutional Review Board of the University of Michigan Medical School (HUM00192898) and registered in ClinicalTrials.gov (NCT05162846).

**Supplementary Information:**

The online version contains supplementary material available at 10.1186/s13063-023-07125-2.

## Administrative information

Note: the numbers in curly brackets in this protocol refer to SPIRIT checklist item numbers. The order of the items has been modified to group similar items (see http://www.equator-network.org/reporting-guidelines/spirit-2013-statement-defining-standard-protocol-items-for-clinical-trials/

**Table:** 

Title {1}	The Michigan Genetic Hereditary Testing (MiGHT) study’s innovative approaches to promote uptake of clinical genetic testing among cancer patients: a study protocol for a 3-arm randomized controlled trial
Trial registration {2a and 2b}.	ClinicalTrials.gov ID: NCT05162846
Protocol version {3}	October 14, 2022
Funding {4}	This work is supported by the NIH NCI (U01CA232827), Principal Investigators (mPIs) Stoffel, Griggs, and Resnicow. Gerido is supported by the National Institutes of Health grant T32 HG010030 (University of Michigan ELSI Research Training Program).
Author details {5a}	1-University of Michigan School of Public Health, Ann Arbor, MI 2-Rogel Cancer Center, University of Michigan, Ann Arbor, MI 3-University of Michigan School of Medicine, Ann Arbor, MI 4- Department of Internal Medicine, University of Michigan, Ann Arbor, MI
Name and contact information for the trial sponsor {5b}	**NIH/NCI Program Official**: Erica S Breslau, PhD, MPH **Email**: breslaue@mail.nih.gov **Phone**: 240 276 6773
Role of sponsor {5c}	The sponsor played no part in study design; collection, management, analysis, and interpretation of data; writing of the report; and the decision to submit the report for publication. The content is solely the responsibility of the authors and does not necessarily represent the official views of the National Institutes of Health or National Cancer Institute.

## Introduction

### Background and rationale {6a}

There are more than 16.9 million cancer survivors in the USA, with nearly 1.9 million new cancers diagnosed each year. Although most cancers are sporadic, germline genetic variants are implicated in 5–10% of cancer cases. Clinical genetic testing identifies pathogenic germline genetic variants associated with hereditary cancer syndromes. An estimated 20% of cancer patients have a family history of cancer, and a subset of these developed their cancers as a result of inherited pathogenic variants in genes associated with cancer susceptibility. Several of these genes are associated with well-known hereditary cancer syndromes, such as *BRCA1/2* for hereditary breast and ovarian cancer (HBOC), *TP53* for Li-Fraumeni syndrome, and *MLH1*, *MSH2*, *MSH6*, *PMS2*, and *EPCAM* for Lynch syndrome. Importantly, most individuals with genetic susceptibility remain undiagnosed. Epidemiological studies have estimated the prevalence of HBOC in the general population to be 1 in 400 [1] but more recent exome research has suggested an even higher prevalence of 1 in 139 [2]. For Lynch syndrome, which is the most common inherited form of colorectal cancer, the general population prevalence is approximately 1 in 279 [3].

Germline genetic testing identifies individuals with cancer predisposition syndromes and supports the use of personalized strategies for cancer prevention, early detection, and/or targeted therapy [4, 5]. The germline genetic testing results carry implications for not only the cancer patient’s own treatment, but also the medical management of their family members [6]. There is a growing demand for cancer genetic services, yet genetic counseling and genetic testing remain underutilized (Bednar et al., 2020). As a result of the increasing number and decreasing cost of genetic tests and the expansion of genetics and genomics into mainstream medicine the demand for genetic counseling services has outpaced the workforce [7, 8]. Lack of access directly impacts treatment options, outcomes, screening for other malignancies, and assessment of at-risk family members [9].

Barriers to accessing genetic testing are multi-tiered. Substantial patient-level barriers to genetic counseling and testing persist, including limited knowledge, financial concerns, competing demands on patients at the time of diagnosis, fear of insurance discrimination, emotional distress, uncertain benefit, time commitment, lack of knowledge about genetic counseling or testing, discouragement by family members, and personal fear [10]. Provider-level barriers may relate to limited knowledge of genomic medicine, insufficient information to assess cancer risk and refer to genetic counseling and testing, and challenges communicating the complexity of genomic medicine adds to cancer care. Population-level barriers to the knowledge of and access to genetic testing have been found among racial/ethnic minorities, people for whom English is a second language, patients with public insurance, and rural communities. These communities have been underserved persistently [11, 12]. Leaders in the cancer genetics community emphasize the importance of developing new models for providing genetics education and counseling to patients who are considering clinical genetic testing for cancer susceptibility [13] and multilevel approaches to overcome barriers to uptake of genetic testing are an area of focus [14]. Given the rapidly expanding indications for genetic testing to guide oncologic treatment decision-making, alternative ways to deliver cancer genetics services (including telehealth, point-of-care, and direct-to-consumer clinical genetic testing) are being employed to expand access [15] and may include digital interventions and counselors without formal education in genetics.

### Objectives {7}

The Michigan Genetic Hereditary Testing (MiGHT) study is a pragmatic randomized controlled trial designed to increase the utilization of genetic testing among eligible cancer patients by addressing health education and behavior barriers (NIH/NCI U01CA232827). While digital health tools and telephone-based coaching have been successful in motivating behavior change across a wide range of health issues, these strategies have not yet been integrated into interventions for facilitating care delivery for patients at risk for hereditary cancer syndromes. The MiGHT study represents a patient-centered approach to increasing the uptake of genetic testing with interactive, web-based technology. Led by our team at the University of Michigan Rogel Cancer Center, the study is conducted in collaboration with the Michigan Oncology Quality Consortium (MOQC), a state-wide network of nearly 90% of medical and gynecologic oncology practices, predominantly community practices throughout the state, and the Michigan Department of Health and Human Services (MDHHS). The MDHHS Cancer Genomics Program was funded through a grant from the Centers for Disease Control and Prevention to increase awareness of genetic testing and counseling and to provide information about genetic resources for patients and health care providers in the state of Michigan (Cooperative Agreement #5U38GD000054).

The primary objective of this three-arm randomized clinical trial is to test the efficacy of two patient-level behavioral interventions on uptake of cancer genetic testing. The two interventions are (1) a virtual genetics navigator with tailored content and, (2) motivational interviewing by genetic health coaches. We have two primary hypotheses concerning the independent comparisons of the active intervention arms 2 and 3 to the usual care (UC), Arm 1:

- *Hypothesis 1* – Arm 2 – a virtual genetics navigator (VGN), will increase the proportion of patients completing genetic testing compared to UC.
- *Hypothesis 2* – Arm 3 – motivational interviewing-based telephone counseling with a genetic health coach (GHC) will increase the proportion of patients completing genetic testing compared to UC.

Secondary objectives are to assess the barriers and motivators for genetic testing of testing uptake and understanding for whom the intervention works (moderators).

### Trial design {8}

A three-arm, randomized control trial will be conducted with participants randomly assigned to either of the two intervention arms (Virtual Genetics Navigator and Genetics Health Coach) or to the control arm (Usual Care). We will prospectively evaluate the noninferiority of the effectiveness of health education delivered with the support of a virtual genetics navigator or motivational interviewing-based telephone coach in comparison to usual care to increase the uptake of genetic testing for hereditary cancers.

## Methods: participants, interventions, and outcomes

### Study setting {9}

Study participants will be identified through oncology practices participating in the MOQC, a physician-led state-wide collaborative quality initiative that includes 68 academic and community oncology practices whose members represent over 90% of the medical and gynecologic oncologists in Michigan.

### Eligibility criteria {10}

Oncology patients are eligible to participate in the MiGHT study if they (1) are 18 years of age or older, (2) can speak and read in English, (3) have access to a telephone and the internet, and (4) self-report a diagnosis of breast, ovarian, prostate, endometrial, pancreatic, or colorectal cancer that meets the National Comprehensive Cancer Network (NCCN) criteria for genetic testing. Personal and family history of cancer will be self-reported through the Family Health History Tool (FHHT). The FHHT is a web-based survey delivered to potential participants by email or SMS/Text which elicits detailed information about family history of cancer (cancer type and age at diagnosis) in first- and second-degree relatives and calculates a score predicting the probability of Lynch syndrome (PREMM5) [16]. Individuals with breast, ovarian, prostate, endometrial, pancreatic, or colorectal cancers that meet the MiGHT study eligibility criteria, which have been adapted from NCCN criteria for genetic testing (Table 1) [17]. Potential participants will be contacted by the study team by email or telephone to provide information about the clinical trial. Individuals who report having previously undergone clinical germline genetic testing or who have already scheduled an appointment for genetic testing are ineligible. This criterion ensures focus on the main outcome of uptake of clinical genetic testing and barriers to and motivations affecting successful completion in those at increased risk for pathogenic variants.

**Table 1:** Criteria for clinical genetic testing for aim 2 randomized trial (adapted from NCCN)

Cancer type	Criteria (any 1 of the following is sufficient)
**Breast**	i. Diagnosed age< 50 years ii. PREMM risk model score ≥ 2.5% iii. Personal history of triple-negative breast cancer iv. Ashkenazi Jewish ancestry v. Personal history of male breast cancer vi. 1st or 2nd degree relative with ovarian cancer, pancreatic cancer, breast cancer diagnosed under 50, triple-negative breast cancer, or male breast cancer
**Colorectal**	i. Diagnosed under 50 ii. PREMM risk model score ≥ 2.5%
**Prostate**	i. Diagnosed age< 50 years ii. PREMM risk model score ≥ 2.5% iii. Ashkenazi Jewish ancestry iv. 1st or 2nd degree relative with ovarian cancer, pancreatic cancer, breast cancer diagnosed under 50, or male breast cancer
**Endometrium/uterine**	i. Diagnosed age< 50 years ii. PREMM risk model score ≥ 2.5%
**Ovarian**	No additional criteria needed
**Pancreatic**	No additional criteria needed

### Who will take informed consent? {26a}

The research staff members review the eligibility criteria for potential participants. Potential participants are then contacted by study staff via email or telephone to confirm the eligibility criteria have been met. Once eligibility has been confirmed, the staff member will add them as a user of the MiGHT study platform. Then the system will send an email to the potential participant. The invitation email includes a personalized link to login to the MiGHT study platform, where the individual confirms whether or not they have taken a genetic test or if they have an appointment scheduled to take a genetic test. If the potential participant has neither they will be able to indicate their consent to participate in the study using the consent form displayed within the MiGHT study platform.

### Additional consent provisions for collection and use of participant data and biological specimens {26b}

N/A, we have no additional consent provisions.

## Interventions

### Explanation for choice of comparators {6b}

Our goal is to increase the uptake of genetic testing in patients who meet clinical criteria for referral but who have not yet been tested or scheduled for testing. For this clinical trial, participants will be randomized to one of 3 parallel arms:

- One control arm - usual care (UC, Arm 1).
- Two intervention arms,
  - Virtual genetics navigator (VGN, Arm 2) and
  - Genetics health coach (GHC, Arm 3)

The rationale for comparing each intervention to usual care is to determine if delivering genetics education virtually or with a health coach is superior to usual care. We consider both interventions less costly than using licensed GCs for which there is a workforce shortage.

### Intervention description {11a}

All participants will have access to the MiGHT study web platform which contains contact information for the study team and links to resources for genetic testing that are publicly available through the Michigan Department of Health and Human Services (MDHHS) Cancer Genomics Best Practices website. The MDHHS publishes lists of genetics service providers in the state of Michigan as well as the phone number for the MDHHS genetics hotline where patients and providers can request more information about obtaining clinical genetics services. The participants randomized to Arm 1 will not have access to any intervention-specific content or functionality.

#### Virtual genetics navigator (VGN) intervention (Arm 2)

Participants randomized to Arm 2 will be directed to the virtual genetics navigator (VGN) module of the MiGHT study platform, The VGN is created to allow participants to navigate through foundational genetics education materials and tailored motivational media encouraging genetic testing. Over the course of the study, participants complete online assessments (at baseline – T0, post-test, 6 months – T1, and 12 months follow-up – T2) to help us tailor content and measure the effect of the interventions. We designed the tailored content to reduce patient-level barriers and broaden the reach, impact, and equity of genetic testing.

The content areas include the following topics: (1) benefits of genetic testing, (2) countering myths about testing, (3) overcoming barriers/fears, (4) education about genetic testing, and (5) how to get genetic testing (e.g., through local genetics specialty clinics, primary care provider or oncologist, direct-to-consumer options). Our message database includes expert-written motivational messages that were iteratively developed through a group review, with content guided by current clinical genetic testing guidelines and motivational interviewing practices. Examples of tailored messaging are provided in Table 2.

**Table 2:** Sample tailored messages

Tailoring item message	Tailoring item message
**Barriers/misconceptions.** This content will appear only for those who answer 4 or higher on a 1–5 agree/disagree scale for each barrier on the list of barriers in the baseline survey and follow-up sessions in Arm 2.	
**Knowing my genetic status would not change … my cancer treatment.**	Excerpt from video: Another benefit of genetic testing is that it gives us a clearer understanding of your future risk for cancer, so we can plan the right follow-up care and monitoring. After your cancer treatment ends, the goal is to keep you from getting cancer again -- either from the same cancer coming back or a new cancer forming. If we know you have a pathogenic variant, we can use medications, screening tests, and sometimes surgery to help prevent future cancer -- or find it in its earliest stages if it does develop. Finding cancer early, when it’s small and has not spread, offers the best chance of treating it successfully.
**If I were found to carry an altered gene, I would feel … Guilty about passing it on**	Excerpt from video: All I could think about after I found out my test results was, “What if I passed this on to my kids?: I love my family and would feel terrible if I was the cause of anything bad in their lives. But you know, I was talking with my doctor about my test results and she reminded me that having a variant is not my fault, I was just born this way. And she said something else that really struck me. She explained how helpful this information would be for my family. At first I didn’t totally understand how me having a pathogenic variant could be a good thing for anyone. But now I get how this information is really useful to share with my family so they can make the best healthcare decisions for themselves. I was actually talking to my daughter the other day and she said she’s planning to see her doctor soon to ask about testing based on my results. I’m so glad I could give my family the information they need to take care of themselves.
**Getting genetic testing would cost me too much money.**	Written message: Good news – genetic testing is usually less expensive than people think! The cost of testing has dropped a lot in the past several years. There are two main ways to pay for testing: Through insurance: ∙ Most insurance plans will cover part or all of the cost if a doctor recommends the test. ∙ Most insurance companies have specific criteria that someone needs to meet based on their personal and family history of cancer. ∙ Insurance may cover one or both types of clinical genetic test (i.e., from a healthcare provider or patient-initiated test). ∙ Before getting tested, ask your clinic or insurance company how much you will have to pay. Self-pay (out-of-pocket): ∙ Instead of using insurance, some people choose to pay out-of-pocket because they are concerned about discrimination or cost. There are laws to prevent this, but genetic test results could affect long-term care, disability, and life insurance coverage. ∙ If you order a patient-initiated test, check with the company to discuss payment options. They may have different payment plans and options for low-cost testing. Whether going through insurance or paying out-of-pocket, patients generally do not have to pay more than $250.
**I would feel anxious while waiting for my results.**	Written message: You’re not alone – in fact, feeling anxious while waiting for genetic test results is very common. The waiting game can be tough but it’s worth it. Whether you get tested through your doctor or order your own test from a company, it typically only takes a few weeks to get the results. These results give you important information that affects your health for the rest of your life. Genetic test results can help you plan your current and future cancer care, as well as cancer prevention strategies. You can also share the results with your family to help them make informed decisions about their own health. Genetic test results may be hard to wait for, but they can empower you to take control of your health and help your family at the same time.
**Values/motivators.** This content will appear in the Arm 2 navigator based on the top 3 personal values they chose at baseline. For each value, tailored videos were created based on whether someone was low or high readiness in the baseline survey and follow-up sessions in Arm 2.	
**Value chosen: good spouse/partner** **Readiness on 0–10 scale: low (0–5)**	When I started my cancer treatment, I was worried for myself but also for my wife. I didn’t know how I’d be able to handle all these appointments and tests while still being there for her. We’ve been together a long time and I’m proud of the relationship we’ve built. It was scary to feel like this cancer might change that. I wanted to do everything I could to get healthy and back to normal. So when my oncologist recommended getting genetic testing, I wasn’t really interested. It seemed like one more test to do and I didn’t really see how it would help. But my wife thought it was a good idea for me to get tested. She brought it up a few different times after that too. I could tell it was important to her. I really value her opinion, so I scheduled a genetic test. Turns out getting tested wasn’t that difficult, and more importantly, the results have been very helpful in planning my treatment and follow-up care. Plus, it’s helped ease some of my wife’s worries. She was right, getting tested has been good thing for my health – and for our relationship.
**Value chosen: good spouse/partner** **Readiness on 0–10 scale: high (6–10)**	I got diagnosed with cancer not long after getting married. We had just finished sending out thank you notes and then, bam, I got the diagnosis – it was a lot of big life changes at one time. In a way, though, it reinforced just how important our relationship is. My husband is a great guy, super supportive and caring. He’s always there for me and came to most of my appointments. But I didn’t want my cancer to take over his life too. When it came to getting genetic testing, I did some research and it became pretty clear it would be helpful information for me to have. I looked online and found a genetic counselor in my area and set up an appointment to get testing. My husband would never say I’m a burden, but I wanted to be proactive wherever I could to take some of this off his plate. Getting my test results has helped me feel more confident in my treatment plan and I felt good having something more concrete to share with my husband, rather than just uncertainty.
**Readiness.** This content will fire based on level of readiness for genetic testing at baseline survey and follow-up sessions in Arm 2 from low to high (0–10).	
**0–3 on 10-point readiness scale**	You’re not sure you’re ready for genetic testing. We designed this website with you in mind – explore all the resources you need to feel confident in your decision.
**4–6 on 10-point readiness scale**	You’re feeling somewhat ready for genetic testing. That’s great! Let’s explore what testing might mean for you and review some helpful resources.
**7–9 on 10-point readiness scale**	You’re feeling pretty ready for genetic testing. That’s great! Let’s explore the benefits of testing and review some helpful resources.
**10 on 10-point readiness scale**	You’re ready for genetic testing. That’s great! We’ve got all the information you need to set up a test.

For example, presentation of the content displayed on the participant’s homepage is tailored and prioritized using the participant’s responses to the baseline survey. If the individual’s baseline survey responses indicated they have specific barriers to genetic testing (e.g., concerns about cost and privacy), then content about overcoming these barriers will appear toward the top of the page. If the individual endorses low readiness for genetic testing, content designed to increase readiness is presented.

Participants will have on-demand access to the VGN and will be allowed to click on content areas that they are interested in learning about. For participants who have not undergone genetic testing, the VGN will ask them to (1) rate their readiness for genetic testing and (2) identify remaining barriers (e.g., “What is holding you back?”). For participants who indicate that they have completed genetic testing, the VGN will provide information about communicating test results to first and second-degree blood relatives.

#### Genetics health coach (GHC) intervention (Arm 3)

Participants randomized to the GHC Arm will access the MiGHT study platform to schedule two coaching telephone calls with a genetic health coach (GHC). The GHC will overcome resistance and knowledge gaps by providing foundational genetics “key facts” and offer resources to help participants access genetic testing services. GHCs are professionals in a health-related field or first-year genetic counseling students who have undergone training in Motivational Interviewing (MI).

MI is a patient-centered communication style [18], which has been used extensively to support autonomous decision-making and positive health behavior changes [19–21]. The MI training of GHCs employed a combination of didactic information and experiential exercises and was delivered by senior author KR and a certified genetic counselor. We deliberately chose to use GHCs rather than certified genetic counselors given the national shortage of clinical genetics professionals and the need to expand reach of clinical cancer genetics services. Our ability to train and hire GHCs showcases a multi-level approach to strengthen the workforce in two ways (1) leverage the growing number of students enrolled in genetic counseling programs nationwide to augment existing education with MI training and (2) tap into other health-related guilds which already have the health communications skills and experience with motivating health behaviors to supplement their current practice with genetics education. GHCs were trained to answer basic questions about genetics and testing but not to give medical advice [22, 23].

Participants will schedule up to two telephone calls (approximately 2 weeks and 3 months after randomization) with a GHC. During each coaching call, GHCs discuss barriers and motivators for genetic testing including a readiness assessment. GHCs and participants work collaboratively to overcome resistance and build motivation to undergo genetic testing. The GHCs will help participants process their own reasons, for or against, testing, including how their current testing status aligns with their goals, and values. After each coaching call, the GHC provides a brief written summary of the discussion (topics covered, things to work on/consider, resources and any other necessary follow-ups). This summary is made available on the participant’s MiGHT study portal. Clinical information such as specific risk assessments or potential screening recommendation changes will not be discussed by the GHCs. All coaching calls will be recorded and the audio files will be stored securely for transcription and further research analysis.

### Criteria for discontinuing or modifying allocated interventions {11b}

Participation is voluntary. Participants may discontinue their interactions with the VGN or GHC at any time.

### Strategies to improve adherence to interventions {11c}

Engaging multiple stakeholders in the development and design of novel health interventions is advocated by researchers [24, 25]. We employed co-design methods to engage and empower patients and health care professionals through the iterative development process of the MiGHT study platform. Using feedback from our advisory board members (described under the “Composition of the coordinating center and trial steering committee {5d}” section) and ethics policy, we identified requirements for reminders and notifications. Reminders to login to the MiGHT study platform to report the status of genetic testing or to attend GHC sessions will be emailed or sent via SMS/text message to improve adherence. Also, participants are offered gift cards following the completion of each survey.

### Relevant concomitant care permitted or prohibited during the trial {11d}

The MiGHT study web platform provides clinically vetted publicly available resources and links to MDHHS and MOQC for all participants to use at their convenience. All participants are encouraged to discuss genetic testing with their healthcare providers and are referred to their treating clinicians for any medical follow-up. If participants choose to undergo clinical genetic testing, this will be coordinated/ordered by the participant’s clinical medical providers or by the participants themselves. No genetic tests will be ordered as part of the MiGHT study, nor will the study team be privy to results from tests completed by study participants.

### Provisions for post-trial care {30}

All participants are encouraged to continue with medical care as prescribed by their healthcare providers and are referred to contact their treating clinicians about medical follow-up. If participants have questions about genetic test results, they will be encouraged to contact their medical team. There is no anticipated harm from participating in this study and we plan to address any unforeseen care needs reflected through participants’ comments and feedback should they arise during the trial.

### Outcomes {12}

The primary outcome measure is participants’ self-reported completion of genetic testing assessed at 6 months after randomization (yes/no). This will be assessed through the follow-up surveys administered via the MiGHT study web platform.

#### Barriers to uptake of genetic testing

Barriers are assessed using 23 items covering multiple domains, which broadly fit under emotional and self-efficacy. The emotional items were adapted from Thompson et al. to assess potential benefits for self/family (informing health behaviors) and potential harms (negative emotional reaction, confidentiality, family worry, guilt, stigma) [26]. The self-efficacy items assess participant confidence in pursuing genetic testing, acting on the information, and communicating results to relatives and were adapted from Katapodi et al. [27]. Each item is scored on a scale from 1 to 5 (1=not at all; 5=extremely). The mean score for each question is calculated to rank the barriers in order of importance. A higher mean score indicates the greater importance of that specific barrier.

#### Motivators to uptake of genetic testing

Motivation for getting tested is measured with an adapted version of the Treatment Self-Regulation Questionnaire (TSRQ) by Levesque et al. Each item is scored on a scale from 1 to 5 (1=not at all; 5=extremely) [28]. The mean score for each question, across all participants who completed genetic testing, will be calculated in order to rank the motivators in importance for purposes of tailoring. A higher mean score indicates the greater importance of that specific motivator. Questions related to motivators are only asked of participants who have not yet completed genetic testing.

### Participant timeline {13}

The participant timeline is shown in

Cancer patients receiving care at MOQC oncology practices will be sent a link to the FHHT by email or SMS/Text 2 weeks prior to their upcoming clinic appointment. One month after completion of the FHHT, eligible individuals with diagnoses of breast, ovarian, prostate, endometrial, pancreatic or colorectal cancers who meet NCCN criteria for genetic testing will be contacted by the study team by email or telephone to invited them to enroll in the clinical trial. After informed consent and completion of the baseline survey (T0), enrolled participants are randomly assigned via the MiGHT study web platform to Arm 1: UC, Arm 2: VGN, or Arm 3: GHC groups. The intervention period lasts 6 months and participants complete surveys to assess the effect of the interventions at 6 months post-intervention (T1) and at 12 months post-intervention (T2).

### Sample size {14}

Based on data compiled by the state of Michigan’s MDHHS Cancer Genomics Best Practices branch, we expect that uptake of clinical genetic testing at 6 months post baseline among participants in the UC group will be 20% or less. With 202 participants per intervention arm, we will have 82% power to find a 14% difference between the VGN mobile-optimized website arm and UC arm, and 99% power to find a 20% difference between the GHC and UC arms. Accounting for attrition, we plan to enroll a total of 759 participants.

### Recruitment {15}

Patients meeting the criteria for genetic evaluation will be contacted by email or by telephone and invited to participate in the clinical trial. Study staff will make up to 20 contact attempts and will direct potential subjects to the MiGHT study web platform. Patients interested in participating in the study will have the opportunity to provide informed consent during a conversation with a study team member or by reviewing the informed consent document through the MiGHT study web platform.

## Assignment of interventions: allocation

### Sequence generation {16a}

After consenting to the study, participants create login accounts on the MiGHT study web platform and complete the baseline survey. Participants are then block randomized and assigned one of the three arms based on the cancer type (strata: breast, ovarian/endometrial, colorectal, pancreatic, prostate) randomly selecting blocks of size 3 or 6 within each stratum. Assignments are made based on the randomized list during the enrollment process after a participant completes the baseline survey.

### Concealment mechanism {16b}

Our study biostatistician prepared the computer-generated random numbers. Then the MiGHT study team information technologists integrated the randomization requirements into the automated functions of the MiGHT study web platform.

### Implementation {16c}

Participants will be informed of the arm to which they have been randomized when they login to the MiGHT study web platform using their assigned username and password. The content displayed will vary by the arm as described in the Interventions section.

## Assignment of interventions: blinding

### Who will be blinded {17a}

No study team members nor participants will be blinded to their randomization arm.

### Procedure for unblinding if needed {17b}

N/A, this study is unblinded.

## Data collection and management

### Plans for assessment and collection of outcomes {18a}

Data are collected through participant-completed surveys, notations from the Genetics Health Coaches, and keystrokes/clicks from the virtual genetics navigator platform. The MiGHT study web platform is used to administer and collect surveys at baseline (upon enrollment, T0) and at 6 and 12 months post-T0.

### Plans to promote participant retention and complete follow-up {18b}

The MiGHT study web platform sends reminders (by email/SMS text) to participants encouraging them to complete the surveys. Participants receive incremental electronic gift card incentives to enroll and to remain in the study. For completing the baseline survey (T0), participants receive $10, for the 6-month survey (T1) they receive $15, for the 12-month (T2) they receive $25. Also, the research team meets regularly to address issues that may impact participant retention.

### Data management {19}

Data for this trial are collected through two sources:

1. *Extraction from the family health history tool (FHHT).* The FHHT is used by MOQC practices to securely collect a comprehensive personal and family cancer history (HUM00180616). The extracted data is use to screen potential participants for eligibility.
2. *The MiGHT study platform* is a secure web application that has seamless integration with Qualtrics surveys. The platform collects data related to logins, page views, and paradata (clicks/keystrokes). In addition, all participants use the platform to complete surveys (baseline, 6- and 12-month follow-ups), participants randomized to the VGN enter data to indicate their progression toward (scheduled appointment) or uptake of genetic testing, whereas participants randomized to GHC will have data collected via semi-structured reports submitted by the coach after each session. The GHC report includes close-ended questions and an unstructured field to provide a written summary of the discussion (topics covered, things to work on/consider, and any other necessary follow-ups).

### Confidentiality {27}

All participant data will be housed in the MiGHT study platform stored in HIPAA-compliant study databases hosted on secure, encrypted servers. No identifiable information about participants will be shared beyond the study team.

### Plans for collection, laboratory evaluation, and storage of biological specimens for genetic or molecular analysis in this trial/future use {33}

N/A, no biological specimens are collected within this study.

## Statistical methods

### Statistical methods for primary and secondary outcomes {20a}

Logistic regression will be used to compare the proportion of participants who complete genetic testing at 6 months between the two active intervention arms with UC. The model will include variables for intervention, cancer type, age, and time since diagnosis (over or under 1 year). As a secondary analysis, we will control for a potential “dosage” effect for the VGN and GHC treatments by including a covariate for dosage. Dosage here is defined as 0 for the UC group, the number of times the website is accessed for the mobile-optimized website group (log-ins), or the number of health coach encounters completed (0,1, 2) for the GHC group.

Secondary analyses will investigate survey data; outcomes will be assessed using linear mixed models. Linear mixed models use all available measurements allowing participants to have an unequal number of observations and produce unbiased parameter estimates as long as the missing observations are missing at random (MAR). The model will include fixed effects for time, indicators for treatment (VGN, GHC, and reference category of UC), and treatment-by-time interactions, cancer type, age, and time since diagnosis (over or under 1 year). Random effects for the intercept and time with an unstructured within-person correlation structure for the residual errors will be specified. Model diagnostics will be used to determine the suitability of more parsimonious (e.g., autoregressive) correlation structures and nonlinear effects for time.

Potential effect modifiers of interest will be entered as interaction terms with the intervention arm. Where interaction terms are significant stratified analyses of outcomes will be performed. For example, if the impact of either the mobile-optimized website or MI counseling differs significantly by gender, we will stratify results for men and women.

### Interim analyses {21b}

N/A, no interim analyses have been identified at this time.

### Methods for additional analyses (e.g., subgroup analyses) {20b}

N/A, no additional analyses have been identified at this time.

### Methods in analysis to handle protocol non-adherence and any statistical methods to handle missing data {20c}

We closely follow the regulatory documentation and reporting process that are strictly implemented by the NIH and U-M IRB for any non-adherence and deviations. All randomized individuals will be analyzed via an intent-to-treat approach. We will work to prevent missing data by the recruitment and retention strategies. The amount and patterns of missing data and its associations with other variables (in particular with the intervention category) will be explored so that an appropriate statistical method for analysis can be used. If the data is missing at random (missing outcomes can be predicted from other observed variables), we will use multiple imputation to handle sporadic missing at random outcomes. Multiple imputation by chained equations will be used with 100*fraction of incomplete cases number of imputations. Results will be combined using Rubin’s rules. In case of non-ignorable missing (missing not at random) data, sensitivity analyses will be performed using pattern mixture or selection models to evaluate the robustness of our conclusions to a range of sensible conditions.

### Plans to give access to the full protocol, participant-level data, and statistical code {31c}

Study team members at the University of Michigan will have access to the deidentified final trial dataset. Long-term storage of de-identified data will be hosted on secured servers, per the data management plan approved by the IRB. Third parties interested in using the final dataset to study related topics may request access and permission from the multiple PIs. Also, permission is required for any publications or dissemination effort. Permission will be granted on a case-by-case basis and with full consideration of the NIH and U-M IRB guidelines.

## Oversight and monitoring

### Composition of the coordinating center and trial steering committee {5d}

The MiGHT study team meets weekly (including the 3 principal investigators, co-Investigators with expertise in motivational interviewing, clinical genetics, behavioral interventions, and members of the Center for Health Communications Research overseeing the web platform and data management). At the study’s inception, a Community and Patient Advisory Board was convened to maintain continuous stakeholder involvement throughout the study. The Advisory Board consists of eight patients/caregivers, two oncologists, two nurses, one administrator, three genetic counselors, and one representative from the MDHHS. The MiGHT study team and Advisory Board meet quarterly, to discuss progress on the study and to obtain feedback on interventions and educational materials as part of our iterative design process.

### Composition of the data monitoring committee, its role and reporting structure {21a}

The Data Safety and Monitoring Committee for the MiGHT study includes 2 internal members (University of Michigan Director of Population Sciences and a second biostatistician) and 3 external members (2 oncologists and one genetic counselor from 3 different academic medical centers outside Michigan).

The study team meets with the DSMB every 6 months or more frequently depending on the activity of the protocol. Topics for discussion include matters related to the safety of study participants (SAE/UaP reporting), validity and integrity of the data, enrollment rate relative to expectations, characteristics of participants, retention of participants, adherence to the protocol (potential or real protocol deviations) and data completeness. At these regular meetings, the protocol-specific Data and Safety Monitoring Report form will be completed and signed by the Principal Investigator or by one of the co-investigators.

Data and Safety Monitoring Reports will be submitted to the University of Michigan Rogel Cancer Center Data and Safety Monitoring Committee (DSMC) every 6 months for independent review.

### Adverse event reporting and harms {22}

The potential risks of this project are anticipated to be minimal with important safeguards in place to protect the welfare of study participants. It is possible that participants may experience some emotional discomfort when thinking about family cancer diagnoses and potential implications for their relatives. However, at the beginning of the survey, we will stress that a participant can stop the surveys, VGN, or GHC sessions at any time if they feel uncomfortable. Our MiGHT study team includes certified genetic counselors, clinical and research psychologists, and practicing physicians who can provide advice and/or facilitate clinical interventions should the need arise. Any adverse events resulting from research procedures will be reported to the IRB and DSMB per institutional guidelines.

### Frequency and plans for auditing trial conduct {23}

The principal investigators convene weekly meetings with the research team to review the progress of the study, recruitment and enrollment status, and identify any adverse events, which may be anticipated or unanticipated. Subject accruals, as well as data and safety monitoring summary reports are submitted to the IRB as part of the annual renewal approval process and to the NIH with the annual progress report.

### Plans for communicating important protocol amendments to relevant parties (e.g. trial participants, ethical committees) {25}

If amendments to the protocol are required, these will be reviewed by the principal investigators and submitted to the IRB for approval prior to implementation. A copy of the revised protocol will be shared with the research team. Any deviations from the protocol are fully documented, reported to the IRB, and updated in the clinical trial registry.

### Dissemination plans {31a}

Trial results will be presented at local, national, and international meetings and disseminated through peer-reviewed publications. We have ongoing meetings with participating MOQC practices. We will present study results at national and international meetings to aid the dissemination of both positive and negative findings. If the MiGHT study interventions are effective, results could inform future MDHHS policy, resources, and tools regarding genetic testing and counseling for hereditary cancer syndromes.

Study progress and findings will be recorded periodically on ClinicalTrials.gov.

## Discussion

The MiGHT study addresses important gaps in our ability to increase the uptake of genetic testing by testing two scalable interventions. The MiGHT interventions deliver an innovative approach for engaging with a state-wide network of oncology practices and their patients in personalized ways. Our MI-based tailored messages and coaching demonstrate how population-level interventions are still able to be patient-centered. The virtual genetics navigator explores how technology may be used to extend the reach of clinical genetics services. Patients are individuals with different values, health histories, and experiences. The interventions developed for the MiGHT study address key barriers and motivators. In this

Over the next three years of the study, we have the opportunity to investigate two methods of delivering personalized genetics education that amplifies individual motivators. By supporting patients most at risk for hereditary cancer with virtual tools and trained genetics health coaches, we hope to address critical workforce shortages, patient education needs, and disparities in the uptake of genetic testing**.**

## Trial status

The recruitment for this 3-arm RCT began February 2, 2022, and will continue until the cohort has accrued, which is anticipated February 2, 2025 (36 months). We plan to complete the follow-up surveys by March 1, 2026 (48 months). The pandemic (COVID-19) has placed a considerable strain on healthcare services, communities, and patients and has presented challenges to study roll-out and delayed participant recruitment. Many smaller oncology practices have limited resources and staff to ensure that their patients are aware of the study and to follow up. This has significantly delayed our research activities and publications.

The study protocol date: Initial approval January 31, 2022; Current version October 14, 2022.

## Supplementary Information

**Additional file 1.**

## Data Availability

The research team members at the University of Michigan will have access to the trial dataset that is deidentified once the trial is completed. The final dataset will be available for researchers who are interested in the related topics after the research team has disseminated the main findings of the research aims. Permission from the PI is required for any publications and dissemination effort.

## Abbreviations

COVID-19: Coronavirus disease 2019
ELSI: Ethical, Legal, and Social Implications
FHHT: Family Health History Tool
GHC: Genetics Health Coach
HBOC: Hereditary breast and ovarian cancer
MDHHS: Michigan Department of Health and Human Services
MiGHT: Michigan Genetic Hereditary Testing
MI: Motivational interviewing
MOQC: Michigan Oncology Quality Consortium
NCCN: National Comprehensive Cancer Network
NCI: National Cancer Institute
NIH: National Institutes of Health
PREMM5: Clinical prediction algorithm that estimates the cumulative probability of an individual carrying a germline mutation in the MLH1, MSH2, MSH6, PMS2, or EPCAM genes
RCT: Randomized controlled trial
VGN: Virtual genetics navigator
