# Supplementary material for: The Michigan Genetic Hereditary Testing (MiGHT) study’s innovative approaches to promote uptake of clinical genetic testing among cancer patients: a study protocol for a 3-arm randomized controlled trial
Source: Trials. 2023 Feb 10;24:105. doi: 10.1186/s13063-023-07125-2 (PMC9911941; doi:10.1186/s13063-023-07125-2)
Supplement: Supplementary file 1 — Additional file 1. [file 13063_2023_7125_MOESM1_ESM.pdf]

Welcome

to the MiGHT (Michigan Genetic Hereditary Testing) Study

We appreciate your interest in joining the MiGHT study

MiGHT
Michigan Genetic Hereditary Testing Study

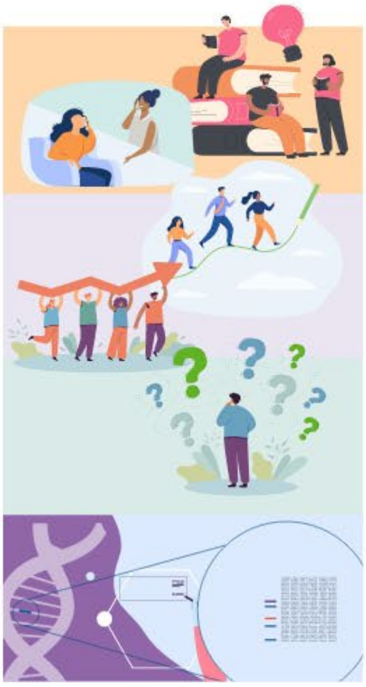

You have the power to help yourself, your family, and others across Michigan.

This study will help you and your family learn about risk for inherited cancer. Findings from the study will be used to help other families in Michigan who have a history of cancer.

If you join the MiGHT study, you will:

- Take 3 surveys over the course of 1 year
- Earn **$50** if you complete all 3 surveys
- Receive phone calls with a genetic health coach **or** access to an online tool about inherited cancer

Find out if this study is right for you:

What is Cancer Genetic Testing?

Genes are made up of DNA and are inherited from each parent. Sometimes genes have changes that can increase your risk for getting certain diseases, including cancer. These changes are known as pathogenic (disease-causing) variants. Blood or saliva tests can look at your DNA to see whether you have any pathogenic variants. This is what we refer to as "cancer genetic testing" in the next questions.

Have you ever had cancer genetic testing?

- ◯ Yes
- ◯ No

Do you have an upcoming appointment scheduled to get cancer genetic testing?

- ◯ Yes
- ◯ No

Next

Survey details

Welcome

Thank you for participating in the MiGHT (Michigan Genetic Hereditary Testing) Study

About the MiGHT Study

Please visit info.mightstudy.org to learn more or email MiGHTStudy@med.umich.edu with questions.

You will receive **another survey approximately 6 months from enrollment** in the study.

Talking with your doctor

If you choose to talk with your doctor about genetic testing, some people find it useful to share a letter to start this conversation. If you want to do this, you can print out a letter we created for you that will help explain the MiGHT study.

Resources for more information about hereditary cancer and genetic testing

MDHHS

Cancer Genomics Program

michigan.gov/mdhhs/

The Cancer Genomics Program works to reduce the impact of hereditary cancer on the people of Michigan and their families.

Hereditary Cancer Resources for Patients and Families

michigan.gov/mdhhs/

Includes information on hereditary cancer, family history, cancer genetic counseling and testing, and cascade screening.

Michigan Department of Health and Human Services Cancer Hotline

Call 1-866-852-1247 or email genetics@michigan.gov

Staff are available Monday - Friday from 9am - 4pm. They can help you find a genetics specialist near you.

They can also answer questions about:

- Hereditary cancer syndromes such as Hereditary Breast and Ovarian Cancer syndrome or Lynch syndrome
- Cancer genetics clinic locations
- Red flags for hereditary cancer
- Available resources

Other Michigan and National Resources

Michigan Genetics Resources Center: Directory of Cancer Genetic Services Providers

migrc.org/providers/

A directory of clinics providing genetic counseling and test coordination in Michigan.

National Directory of Genetic Counselors

findageneticcounselor.nsgc.org

A directory of genetic counselors across the US and Canada. Offers an option to search for in-person or telehealth counselors.

You're feeling somewhat ready for genetic testing.

That's great! Let's explore what testing might mean for you and review some helpful resources.

We recommend starting here:

Your ***Concerns***

Let's look at your specific worries about testing. We want to help address what's holding you back.

Read More

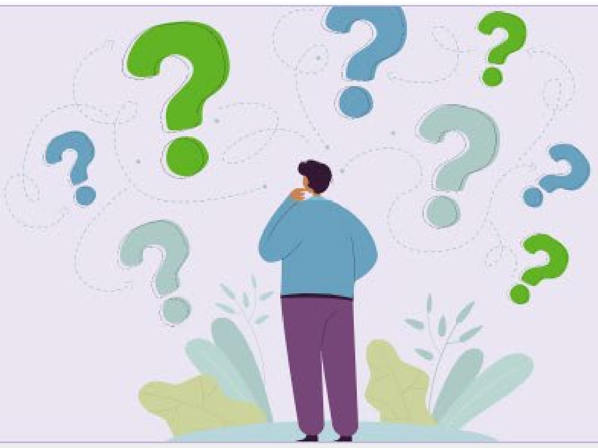

Then you can check out:

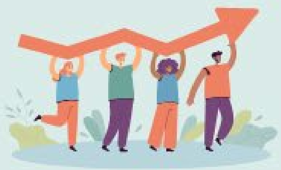

Your ***Motivators***

You've told us what's important to you in life. We want to help you understand the specific benefits of testing for you.

Read More

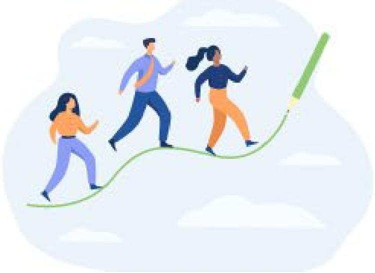

Get Genetic Testing

Find genetic testing and counseling resources near you.

Read More

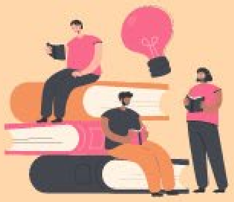

Genetic Testing ***Facts***

Learn how testing works and get the accurate information you need.

Read More

Testing panel

Get Genetic Testing Key Benefits

Upcoming Call

1/30/2023 at 03:00 PM

Reschedule

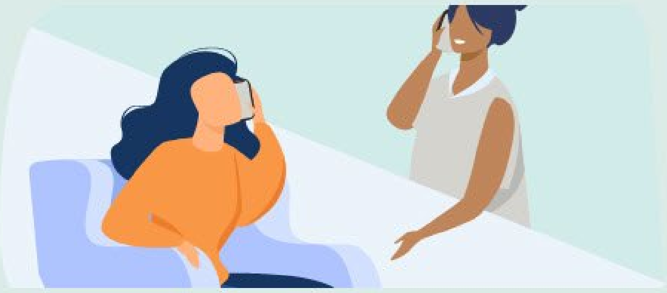

Your Health Coach will call you to talk about genetic testing.

You can choose to discuss questions or issues most important to you.

- Your family or personal history of cancer
- Your concerns about getting genetic testing
- Your core values
- Your motivators to getting genetic testing
- Benefits of genetic testing
- How genetic testing works and how to get tested

Get Genetic Testing

Find genetic testing and counseling resources near you.

Read More

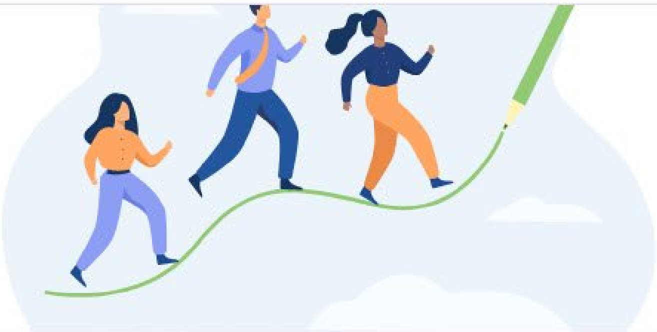

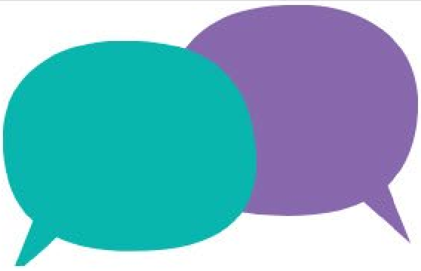

Completed Sessions

After your first coaching session, your summary will be available.

Helpful links from your Health Coaches

Your counselor can send you helpful links. They will appear here on your home page.

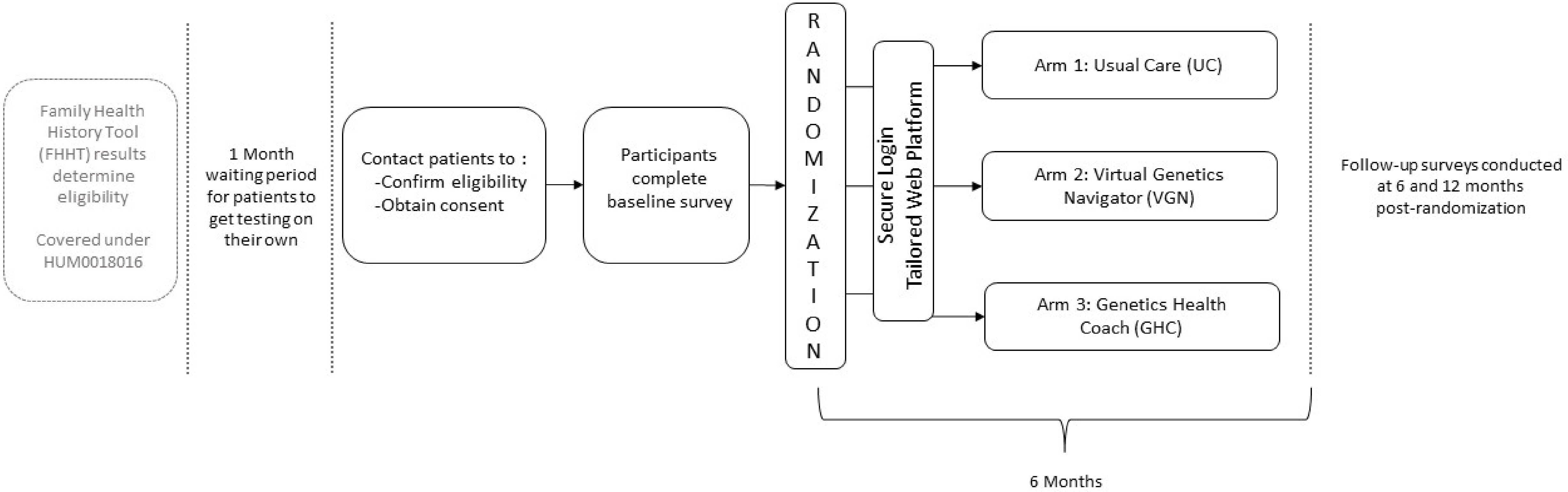
Family Health History Tool (FHHT) results determine eligibility
Covered under HUM0018016
1 Month waiting period for patients to get testing on their own
Contact patients to :
-Confirm eligibility
-Obtain consent
Participants complete baseline survey
RANDOMIZATION
Secure Login
Tailored Web Platform
Arm 1: Usual Care (UC)
Arm 2: Virtual Genetics Navigator (VGN)
Arm 3: Genetics Health Coach (GHC)
6 Months
Follow-up surveys conducted at 6 and 12 months post-randomization
